# Obstructive shock in a 47 year old female with a deep venous thrombosis due to intravascular leiomyomatosis: a case report

**DOI:** 10.4076/1757-1626-2-8159

**Published:** 2009-07-22

**Authors:** Marcus William Butler, Abraham Sanders

**Affiliations:** Division of Pulmonary and Critical Care Medicine, New York Presbyterian Hospital-Weill Cornell Medical College520 East 70^th^ Street, Starr 505, New York, NY 10021USA

## Abstract

**Introduction:**

Intra cardiac tumours and tumour thrombi can present in a manner resembling a massive pulmonary embolism. Intravascular leiomyomatosis with intracardiac extension is one such rare tumour. Survival from obstructive shock in this condition has not been previously reported.

**Case presentation:**

A case is presented of a female who presented with recurrent syncope, cyanosis and then circulatory shock. An intravascular and intracardiac mass was suspected. Due to refractory shock, she ultimately underwent single stage median sternotomy and exploratory laparotomy, with excision of an intravascular leiomyoma.

**Conclusion:**

Intravascular leiomyoma with intracardiac extension should be suspected in the differential diagnosis of a female with a history of uterine fibroids or hysterectomy and presenting with right heart obstructive symptoms.

## Introduction

Intracardiac tumors and tumor thrombi are rare causes of syncope, hypotension and pulmonary embolism, and can present diagnostic and therapeutic challenges for physicians and surgeons involved in their care. While atrial pedunculated masses are usually atrial myomas, myxomas are less common in the right atrium than the left, and other differential diagnoses have to be entertained including venous thrombus or thromboembolism-in-transit, metastases, and primary malignant cardiac tumors such as the cardiac sarcomas [1]. Here we have described a rarer but well reported cause of an intracardiac mass, an intravascular leiomyoma with intracardiac extension, with an unusual clinical presentation.

## Case presentation

A 47 year-old Asian female was transferred to the authors’ institution from another hospital with a subacute history of shortness of breath, bilateral lower extremity swelling, pleuritic chest pain, cyanosis, episodic loss of consciousness and hypotension. Two years prior to admission she had underwent an abdominal hysterectomy for uterine fibroids, which were complicated by symptomatic anemia from associated menorrhagia. Two months prior to admission, she sustained a brief loss of consciousness, then two weeks before admission she developed gradual onset of pleuritic chest pain and lower extremity edema. One day prior to admission, she complained of some shortness of breath and a non-productive cough. There were no known risk factors or family history of thromboembolism. She had no other significant past medical, surgical or obstetric history. She was a non-smoker, non-alcoholic and denied use of illicit substances. She had no history of drug allergy.

On the day of admission, she developed dizziness at home and fell to the ground. Her family helped her to bed wherein she had a witnessed four-minute period of loss of consciousness. She was taken by ambulance to an outside hospital where she appeared cyanotic and hypotensive with a weak pulse, was immediately sedated, endotracheally intubated and started on intravenous norepinephrine. Physical examination, prior to intubation, revealed a dusky blue skin suggestive of cyanosis. She had a tachycardia with pulse rate of 110 per minute, hypotension with systolic blood pressure of 70 mmHg and tachypnea with respiratory rate of 34 breaths per minute. There was teeth clenching and hypersalivation. An electrocardiogram and serum troponin levels were normal, her initial arterial blood pH was 7.25 with a PCO_2_ of 2.93 kPa (22 mmHg) and a PO_2_ of 67.46 kPa (506 mmHg) on an inspired fraction of oxygen of 1.0. The leukocyte count was 12.9 x 10^3^ /μL with 81% neutrophils and 16.2% lymphocytes. Other hematologic and coagulation parameters were within the normal range. The serum bicarbonate was 14.6 mmol/L while other serum chemistries, renal and liver function tests were unremarkable. A computed tomography (CT) scan of the head was normal. A venous duplex ultrasound scan showed the presence of a right lower extremity proximal deep venous thrombosis (DVT) extending to the groin and she was commenced on intravenous standard unfractionated heparin.

**Figure 1.**
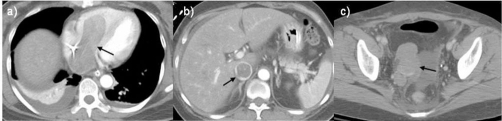
Computed Tomography images. CT of **(a)** the chest, **(b)** abdomen and **(c)** pelvis (soft-tissue gating). **(a)** A large hypodense filling defect is seen, 3.1 cm in diameter, traversing the right atrium to the right ventricle (arrow) with bilateral small pleural effusions and atelectasis. There was no evidence of a pulmonary embolism (images not shown); **(b)** Distension of the inferior vena cava (IVC) with the same tissue density thrombus (arrow) as was seen in **(a)** and with evidence of some linear enhancement, difficult to appreciate here, suggestive of a tumor thrombus; **(c)** A 6.2 x 3.9 cm lobulated pelvic mass (arrow) which was inseparable from the internal iliac vein.

**Figure 2.**
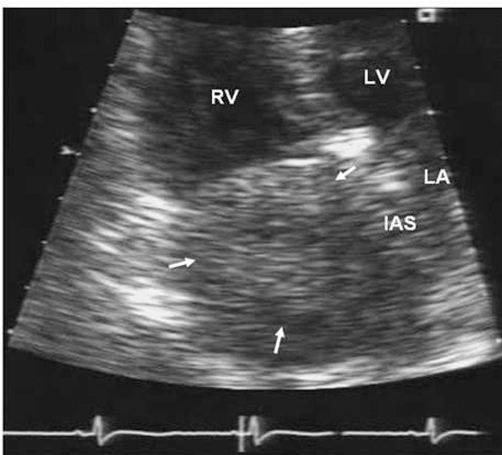
Echocardiographic appearance. Transthoracic echocardiogram apical four chamber view in ventricular diastole shows a 3.5 x 3cm tissue-density echo (arrows) filling the right atrium and reaching the plane of the tricuspid valve annulus. RV, right ventricle. LV, left ventricle. LA, left atrium. IAS, interatrial septum.

Later that day the patient was transferred to our hospital where a pulmonary embolism protocol computed tomography (CT) scan of the thorax and pelvis (Figure 1a, 1b, 1c) and a repeat transthoracic echocardiogram were performed (Figure 2). Because of the positive venous duplex ultrasound finding of a DVT, a D-Dimer was not requested.

**Figure 3.**
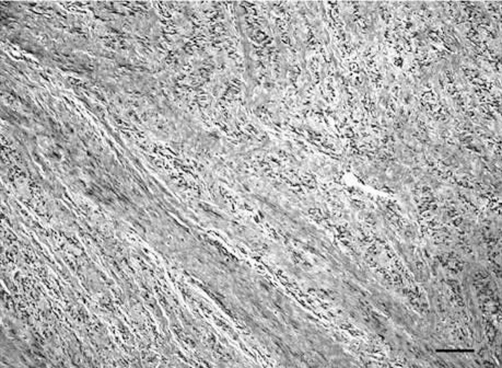
Histologic appearance. High power magnification of a hematoxylin and eosin-stained tissue section from the intracardiac portion of the tumor showing interweaving bundles of proliferating smooth muscle cells with no abnormal mitotic activity. Scale bar = 0.05 mm.

The patient was seen by a pulmonologist who made a differential diagnosis of an intracardiac tumor thrombus versus a thromboembolism-in-transit, and advised a surgical opinion. Because of delays in obtaining a concensus on appropriate surgical management from the cardiothoracic, vascular and gynecologic surgeons together with the need for further imaging studies, ongoing hypotension, presumed transient complete occlusion of the tricuspid valve and a confirmed deep venous thrombosis, the patient was initially thrombolysed intravenously with 100 mg of tissue plasminogen activator to treat any life-threatening component of venous thrombosis. Following this, the patient’s hemodynamic indices improved only slightly. The next day, the patient underwent a joint procedure with single stage median sternotomy and exploratory laparotomy, extraction of an inferior vena cava, common iliac vein and right atrial tumor thrombus, and included bilateral salpingo-oophorectomy. The histopathology of the tumor was consistent with leiomyoma (Figure 3).

Post-operative transoesophageal echocardiogram and CT showed complete removal of the intravascular lesion with minimal residual pelvic mass. There were no complications and the patient was subsequently taken off pressors, extubated and discharged to follow-up on oral anticoagulation.

## Discussion

Intravenous leiomyoma is a rare smooth muscle tumor arising from either the wall of a uterine vessel or from a uterine leiomyoma [1-3]. The entity was first described in 1896 [1] and the first reports of intracardiac extension were described separately by Durck and Hormann in 1907 [2-4]. To date, there have been at least 121 case reports of intravenous leiomyomatosis with intracardiac extension, though the tumor is usually confined to pelvic veins [5-7]. Right-sided congestive symptoms and syncope due to transient obstruction at the tricuspid valve are the most common intracardiac manifestations [8]. While death from right heart obstruction has been described in 5 cases [2,3,9-11], ours is the first reported case in the medical literature of obstructive shock in a survivor of this rare condition. Other rare manifestations include high output heart failure [12] and massive ascites [13]. Many patients have coexistent uterine leiomyomata [5-7] or have undergone hysterectomy for same [5,8,14].The tumor can present as an intracardiac lesion weeks to years later [5,14]. Though generally benign, it can rarely metastasise to the lung [14].

The aetiology of intravenous leiomyomatosis has been debated; it appears to either represent intravascular extension of uterine leiomyoma or is derived from smooth muscle cells in the vein’s medial layer [5,6,8,14]. The tumor can be entirely free-floating within the vessel lumen or, less commonly, it can have attachments to the vessel or atrial walls [6]. A cytogenetic characterization of the disease has more recently been described, consisting of the karyotype 45, XX, der(14)t(12;14)(q15;q24),-22. These chromosomal aneusomies have been detected at different tumor sites and in different cases of intravascular and uterine leiomyomatosis in separate patients, implying that the intravenous tumor is closely related to uterine leiomyomata pathogenetically [15].

An important differential diagnosis to outrule is a venous thromboembolism in transit, and together with atrial myxomata can represent a diagnostic challenge, given the greater prevalence of these conditions [8,16]. Right heart thromboemboli are essential to exclude, as the mortality is as high as 27%, and 20% of such deaths occur in first 24 hours [17]. The diagnosis of intravenous leiomyomatosis with intracardiac extension is usually made at the time of surgery, but prior imaging can greatly facilitate the exclusion of a thrombus, particularly by echocardiography. Typical appearances of the tumor are an elongated mobile mass extending from the veins of the lower body including the IVC and azygos vein, multiple venous attachments and filling of the venous vessels and right heart chambers [8]. Lack of a pedunculated stalk arising from the atrial wall helps exclude an atrial myxoma [16].

Features suggestive of a thrombus-in-transit include the setting of a post-operative state, indwelling intravascular devices and immobility, with other venous thromboembolism risk factors, and elongated mobile masses of venous casts giving a “popcorn” appearance [8]. In addition, computed tomography (CT) and magnetic resonance imaging (MRI) can give additional valuable information regarding the site and extent of the lesion, to rule out any renal mass suggestive of renal cell carcinoma and to detect associated uterine leiomyomata or pulmonary emboli [14,16]. Nonetheless, the diagnosis of thrombus-in-transit versus a tumor is not always clear on echocardiography or with other imaging [8]. The presence of a DVT in the current case made an embolism-in-transit more difficult to exclude with certainty. Intravenous leiomyomatosis itself can have associated thrombus, though this is unusual [14], and most intravenous leiomyomatosis patients will not be as hemodynamically unstable as the case described herein, and therefore the need for consideration of thrombolytic therapy is less pressing [8].

Treatment of intravascular leiomyomatosis with intracardiac extension is usually surgical requiring complete excision of the tumor. In the past this was mainly done via a separate sternotomy with cardiopulmonary bypass and follow up laparotomy for the subdiaphragmatic lesion but has evolved to a single stage operation [14]. The previous requirement for sternotomy can be avoided by using endovascular techniques combined with transoesophageal echocardiography (TOE) to achieve distal control and prevent embolisation during excision, though bypass should still be available. Careful pre-operative assessment and imaging is vital to success, and may include the imaging modalities mentioned above, including TOE and contrast cavography [14]. Hormonal therapies such as gonadotrophin releasing hormone (GnRH) agonists, radiation and chemotherapy have also been used where surgery is contraindicated and for metastatic disease [8]. Recurrence and metastases can be as late as 15 years and requires long-term follow up including CT thorax and abdomen and serial echocardiography [7,8].

## Conclusion

Intravascular leiomyomatosis is a rare cause of right heart obstructive symptoms but should feature in the differential diagnoses of a right heart mass arising in a female post hysterectomy or with known uterine fibroids. A careful history, physical examination and appropriate diagnostic imaging can rule out other potential diagnoses allowing the physician to refer the patient for definitive surgical treatment.

## Abbreviations

CT: Computed tomography
DVT: Deep venous thrombosis
IVC: Inferior vena cava
MRI: Magnetic resonance imaging
TOE: Transoesophageal echocardiogram
